# AutoEncoder-Based Computational Framework for Tumor Microenvironment Decomposition and Biomarker Identification in Metastatic Melanoma

**DOI:** 10.3389/fgene.2021.665065

**Published:** 2021-05-27

**Authors:** Yanding Zhao, Yadong Dong, Yongqi Sun, Chao Cheng

**Affiliations:** ^1^Department of Medicine, Baylor College of Medicine, Houston, TX, United States; ^2^Institute for Clinical and Translational Research, Baylor College of Medicine, Houston, TX, United States; ^3^Beijing Key Lab of Traffic Data Analysis and Mining, School of Computer and Information Technology, Beijing Jiaotong University, Beijing, China

**Keywords:** biomarker, gene expression profile, SKCM, tumor microenvironment, immunotherapy

## Abstract

Melanoma is one of the most aggressive cancer types whose prognosis is determined by both the tumor cell-intrinsic and -extrinsic features as well as their interactions. In this study, we performed systematic and unbiased analysis using The Cancer Genome Atlas (TCGA) melanoma RNA-seq data and identified two gene signatures that captured the intrinsic and extrinsic features, respectively. Specifically, we selected genes that best reflected the expression signals from tumor cells and immune infiltrate cells. Then, we applied an AutoEncoder-based method to decompose the expression of these genes into a small number of representative nodes. Many of these nodes were found to be significantly associated with patient prognosis. From them, we selected two most prognostic nodes and defined a tumor-intrinsic (TI) signature and a tumor-extrinsic (TE) signature. Pathway analysis confirmed that the TE signature recapitulated cytotoxic immune cell related pathways while the TI signature reflected MYC pathway activity. We leveraged these two signatures to investigate six independent melanoma microarray datasets and found that they were able to predict the prognosis of patients under standard care. Furthermore, we showed that the TE signature was also positively associated with patients’ response to immunotherapies, including tumor vaccine therapy and checkpoint blockade immunotherapy. This study developed a novel computational framework to capture the tumor-intrinsic and -extrinsic features and identified robust prognostic and predictive biomarkers in melanoma.

## Introduction

Melanoma is one of the most aggressive tumors, with about 160,000 newly diagnosed cases worldwide each year (Schadendorf et al., 2015; Torre et al., 2015). Although the 5-year overall survival of metastatic melanoma patients has increased up to over 50% with checkpoint blockade immunotherapy (CBI) (Larkin et al., 2019), there are still about half of the patients who do not respond to current immunotherapy whose prognosis remain poor (Khair et al., 2019). Thus, identifying comprehensive gene signatures that predict the responses to immunotherapy and melanoma patients’ overall survival would facilitate the clinical practices of melanoma patients.

Both the tumor cell-intrinsic and cell-extrinsic factors influence the progression and regression of cancer. Extrinsically, immune cell infiltration is a hallmark of melanoma (Li et al., 2016; Thorsson et al., 2018). Four molecular subtypes of metastatic melanoma patients based on the gene expression have been identified and the immune subtype patients had significantly prolonged overall survival (Jönsson et al., 2010). This tumor immune microenvironment can be largely affected by tumor intrinsic features (L. Yang et al., 2019). Several studies reported the positive association between the number of non-synonymous somatic mutations and the abundance of tumor-infiltrating immune cells (Li et al., 2016; Varn et al., 2017). On the contrary, copy number variation (CNV) presented a negative association with immune cell infiltration in the tumor microenvironment across multiple cancer types (Davoli et al., 2017; Zhao et al., 2019). In addition to the genomic features, the tumor oncogenic pathways play a profound role in regulating the immunosuppressive tumor microenvironment and immune evasion (Hanahan and Weinberg, 2011). MYC, as an important transcription factor, has been reported to cooperate with Ras to exclude the infiltration of immune cells (L. Yang et al., 2019). In line with these findings, it has been shown that melanoma patients with high somatic mutation burden, low CNV, or low oncogenic activation are more likely to benefit from immunotherapy (Snyder et al., 2014; Van Allen et al., 2015; Davoli et al., 2017; Lauss et al., 2017).

In order to comprehensively characterize these cell-extrinsic and cell-intrinsic factors in patients, linear regression-based models have been widely used to identify gene signatures in patients. Zhao et al. identified 25 immune-associated genes to depict the abundance of tumor-infiltrating immune cells (Zhao et al., 2019), and Liao et al. combined the expression of two immune genes, CCL8 and DEFB1, for prognosis prediction (Liao et al., 2020). However, the algorithms based on linear regression ignored the complicated nonlinear relationships and correlations among genes. Currently, only few methods designed nonlinear models to capture the tumor-infiltrating immune cells in the microenvironment but mostly focused on the function of specific immune cell populations (Yoshihara et al., 2013; Varn et al., 2016). Thus, in this study, we proposed an Autoencoder-based computational framework to extract both the tumor-intrinsic and -extrinsic features from gene expression of melanoma samples. By applying this framework to the TCGA metastatic melanoma RNA-seq dataset, we identified a number of interrelated nodes. Many of these nodes are found to be significantly associated with patients’ prognosis. We selected two most prognostic nodes and defined a tumor-intrinsic (TI) signature and a tumor-extrinsic (TE) signature. Using benchmarked experimental data, we validated that the TE signature reflected the immune cell cytotoxicity pathway while the TI signature captured the MYC oncogenic pathway activity. Both signatures were strong predictors for metastatic melanoma patients’ overall survival, even after adjusting for several clinical factors. Moreover, the TE signature could predict the patients’ response to MAGE-A3 and anti-CTLA4 immunotherapy. Our results provided a generic computational framework for tumor-intrinsic and -extrinsic feature extraction and identified potential biomarkers for predicting clinical outcome in melanoma.

## Results

### Overview of the Study

**FIGURE 1:**
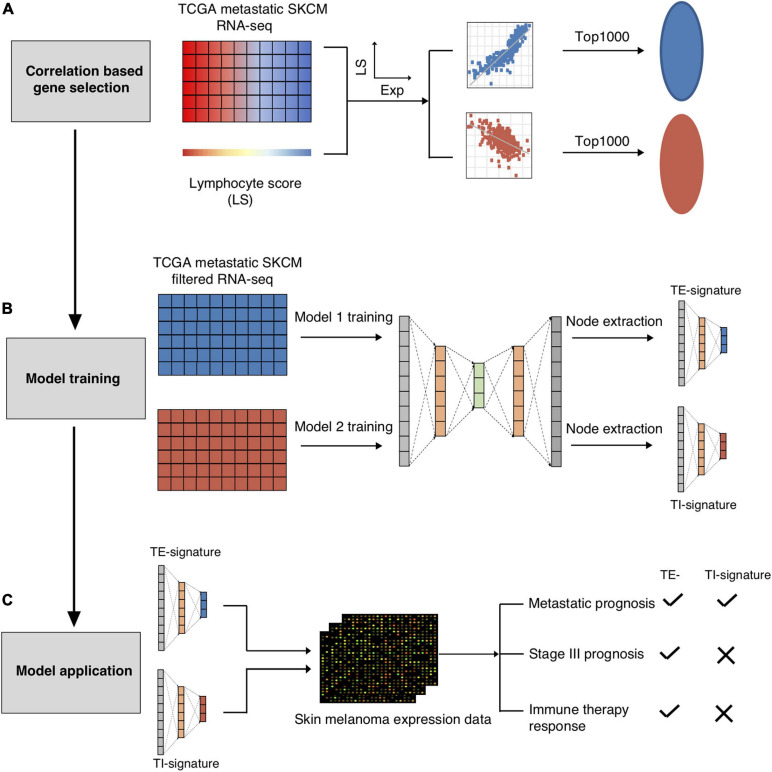
Schematic overview of present study. The TCGA-SKCM metastatic RNA-seq dataset was used to screen out the immune cell and tumor cell related genes. **(A)** The RNA-seq dataset was further split into immune cell related genes expression dataset and tumor cell related gene expression dataset for AutoEncoder decomposition models training. **(B)** Node TE-signature and TI-signature were chosen as the representatives of the immune cell and tumor cell gene expression datasets. **(C)** The trained models were further applied into the independent melanoma gene expression dataset for decomposition. Node TE-signature and TI-signature were then examined for predicting prognosis and immune therapy response.

We extracted the tumor-intrinsic and -extrinsic signals from the gene expression data of metastatic melanoma patients in TCGA and identified a number of interrelated modules (Figure 1). Among these modules, we identified two representatives associated with tumor-extrinsic (TE) and -intrinsic (TI) features, respectively. We further validated that the TE signature reflected the immune cell cytotoxicity pathway while the TI signature indicated the MYC oncogenic pathway activity. Subsequently, we systematically investigated the function of the extrinsic and intrinsic features in melanoma patients’ prognosis and response to immunotherapy, which could be summarized as (1) illustrating the prognostic value of the TE signature and TI signature in metastatic and stage III melanoma patients; (2) developing an integrative model to predict patients’ overall survival; (3) examining the prediction power of the TE signature in immunotherapy; and (4) identifying the association between the TI signature and anticancer drugs.

### Association of the TI and TE Signatures With Molecular and Immunological Features

**FIGURE 2:**
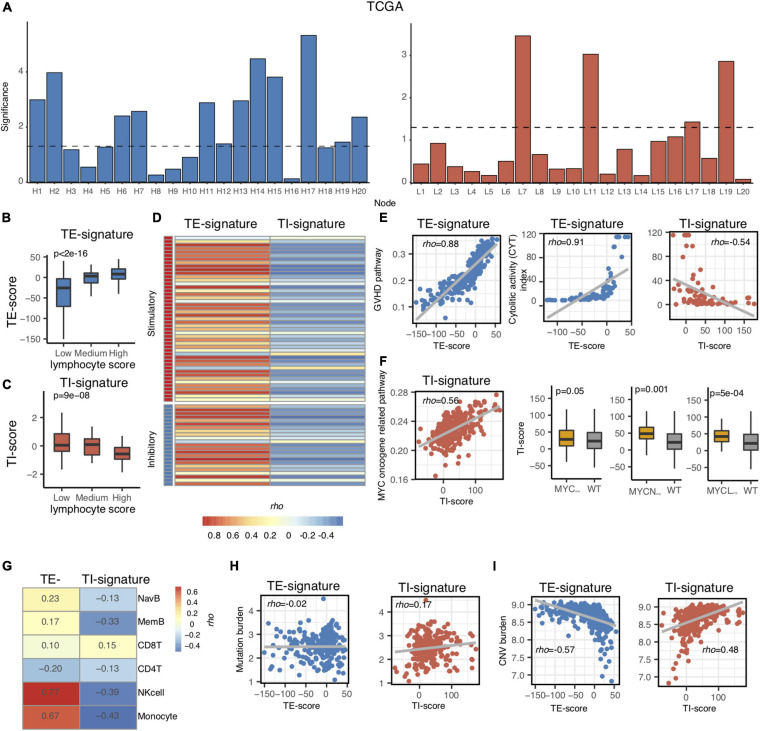
Association of TI and TE signatures with molecular and immunological features. **(A)** Bar plot showing the –log10 (*p*-value) of each node in the TCGA metastatic melanoma dataset. **(B,C)** Boxplot showing the association between TE-signature score and lymphocyte score in **(B)** and between TI-signature score and lymphocyte score in **(C)**. *P*-value was calculated by ANOVA. **(D)** Heat map showing the correlation between immune stimulatory or immune inhibitory gene expression and TE-signature or TI-signature scores. **(E)** Scatterplot showing the correlation between GVHD pathway activity and TE-signature score (left panel) or showing the correlation between CYT index and TE-signature or TI-signature scores (right panel). **(F)** Scatterplot showing the correlation between MYC oncogene pathway activity and TI-signature score. Boxplot indicating the TI-signature scores difference between MYC_amp_ or MYCN_amp_ or MYCL_amp_ and WT. *P*-values were calculated by Wilcoxon rank-sum test. **(G)** Heat map showing the correlation between TE-signature or TI-signature scores and immune cell abundance. **(H)** Scatterplot showing the correlation between Mutation burden and TE-signature (left panel) or TI-signature (right panel) scores. **(I)** Scatterplot showing the correlation between CNV burden and TE-signature (left panel) or TI-signature (right panel) scores. In all scatterplots, the rho was calculated by Spearman correlation.

In total, 40 nodes were acquired (20 nodes from TE-associated modules and 20 from TI-associated modules). An additional feature selection process was performed to select the most clinically relevant nodes. We first examined the prognostic value of each node in the training data (metastatic TCGA SKCM) and chose the TE-signature (H17) and TI-signature (L7) nodes as the representatives for tumor-extrinsic and -intrinsic features given their performances in predicting prognosis (Methods, Figure 2A).

As mentioned in Figure 1, we only chose the genes that were correlated with lymphocyte abundance as the input for training. Therefore, we further validated that the TE signature and the TI signature are associated with lymphocyte abundance (*p* < 2e-16, Figure 2B; *p* = 9e-08, Figure 2C). Additional correlation analyses with immune-stimulatory and inhibitory genes confirmed that the TE signature and TI signature were correlated with the immune microenvironment in the tumor with TE signature presenting a positive correlation and TI signature presenting a negative correlation (Figure 2D). Those evidences showed that the TE signature and TI signature maintained the original correlation structure with the lymphocyte score.

Next, we aimed to explore the pathways that the TE signature and TI signature represent to unravel their biological indications. Based on the pre-ranked GSEA results of the TE signature (Supplementary Table 2), we hypothesized that the TE signature was associated with immune cell cytotoxicity-related pathways. To test this, the pathway activity for each patient was identified using the TCGA metastatic SKCM patients’ expression data of the genes in each pathway of the MsigDB C2 pathway database. The pathway activity of all the pathways in the MsigDB C2 database was then correlated with the TE-signature score for each patient. Shown in Figure 2E, the TE-signature score was correlated with the pathway activity of Graft Versus Host Disease (GVHD), mediated by pro-inflammatory immune components (Henden and Hill, 2015; Kuba and Raida, 2018). The hypothesis was further supported by a strong correlation between the TE-signature score and the cytolytic activity (CYT) index in TCGA metastatic melanoma patients (Rho = 0.91, Figure 2E). To gain insights on the immune cell subtype contributing to this cytolytic activity, the infiltration levels of six major immune subtypes (NK cell, naive B cell, memory B cell, CD8^+^ T cell, CD4^+^ T cell, and monocytes) were correlated with the TE-signature score, which showed that the NK cells having the highest correlation (Figure 2G).

We also explored if the TI-signature score captured similar immune profiles. We found strong negative correlations between the TI signature with the CYT index as well as the infiltration of the six immune cell subtypes (Rho = −0.54, Figures 2E,G), indicating that the TI signature could rather associate with the tumor-intrinsic but not -extrinsic pathways in the TME. Interestingly, the TI-signature score presented a consistent positive correlation with multiple MYC oncogene-related pathways (Figure 2F and Supplementary Table 3). MYC, MYCL, or MYCN amplification-induced MYC pathway activation was reported through many studies (Schaub et al., 2018). Thus, the association between the TI-signature and MYC/MYCL/MYCN amplification status were examined and the results indicated that the TI-signature score represented the MYC pathway in the tumor cells.

Evidences above suggested that the TE signature was associated with immune cell cytotoxicity while the TI signature was associated with MYC pathway activation. These tumor cell-intrinsic and -extrinsic features were largely affected by tumor mutation burden and copy number variation burden (Hanahan and Weinberg, 2011; Chalmers et al., 2017; Taylor et al., 2018). Thus, we further correlated tumor mutation burden and copy number variation burden with both signatures and found that the tumor mutation burden only correlated with the TI-signature score with Rho = 0.17 while the tumor copy number variation burden correlated with both the TE-signature and the TI-signature scores with Rho = −0.57 and Rho = 0.48, respectively (Figures 2H,I).

### TE and TI Signatures Were Predictive of Prognosis in Metastatic Melanoma

**FIGURE 3:**
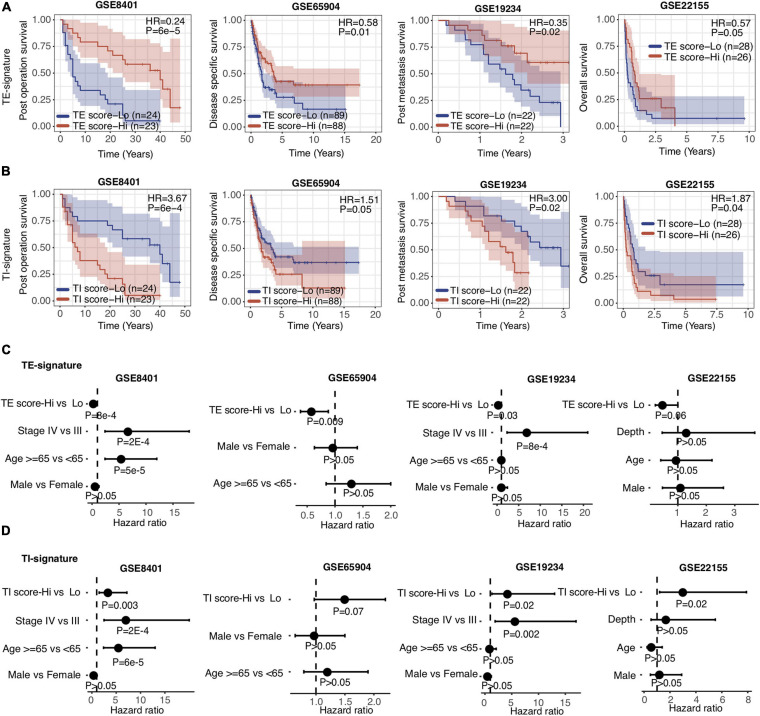
TE signature and TI signature are prognostic in metastatic melanoma. **(A,B)** Kaplan–Meier plots depicting the survival distribution for patients with high (red) and low (blue) TE-signature or TI-signature scores. In Kaplan–Meier plots, *p*-values were calculated using the log-rank test and vertical hash marks indicate censored data. **(C,D)** Forest plot showing hazard ratios and *p*-values of TE-signature score **(C)** or TI-signature score **(D)** and several clinical variables estimated by a multivariate Cox regression model. In all forest plots, HR was presented as the 95% confidence interval, the dotted lines indicate the null association, and the Wald’s test was used to determine statistical significance.

Aforementioned, the TE and TI signatures were chosen based on their prognostic values for metastatic melanoma patients from TCGA, where the TE-signature score associated with better prognosis, yet the TI-signature associated with poor prognosis. The prognosis values of both signatures were further expanded to four other independent metastatic melanoma datasets (GSE8401, GSE65904, GSE19234, and GSE22155). Consistent with the results in the TCGA dataset, patients with higher TE-signature scores had significantly better survival outcomes, while the patients with higher TI-signature scores had worse overall survival (Figures 3A,B). Importantly, the distinctive prognostic values of the TE and TI signature were stable across all the datasets, although each dataset had different patient numbers and collection criteria. To further investigate whether the TE signature and TI signature added additional prognostic values to well-established clinical factors, we applied a multivariate Cox regression model and found that both signatures maintained as predictors for patients’ overall survival even after adjusting for clinical covariates (e.g., tumor pathological stage at diagnosis, patients age and gender) (Figures 3C,D).

### TE Signature Predicted Prognosis in Stage III Melanoma Patients

**FIGURE 4:**
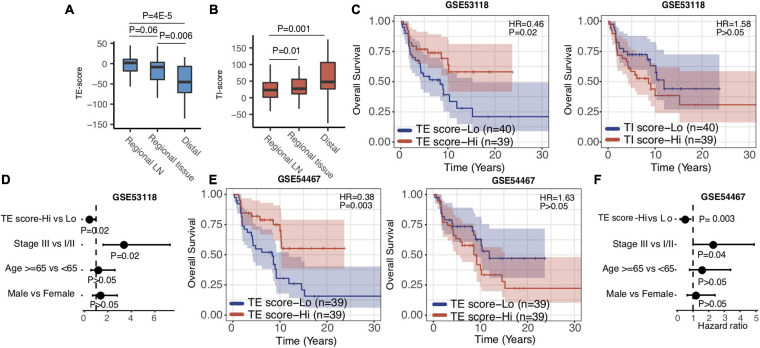
TE signature predicts prognosis in stage III melanoma patients. **(A,B)**. Boxplots indicating the difference of TE-signature or TI-signature scores across different metastatic regions. *P*-values were calculated by the Wilcoxon rank-sum test. **(C)** Kaplan–Meier plots depicting the survival distribution for patients with high (red) and low (blue) TE-signature or TI-signature scores. **(D)** Forest plot showing hazard ratios and *p*-values of TE-signature scores and several clinical variables estimated by a multivariate Cox regression model. **(E)** Kaplan–Meier plots depicting the survival distribution for patients with high (red) and low (blue) TE-signature or TI-signature scores. **(F)** Forest plot showing hazard ratios and *p*-values of TI-signature score and several clinical variables estimated by a multivariate Cox regression model. In Kaplan–Meier plots, *p*-values were calculated using the log-rank test and vertical hash marks indicate censored data. In all forest plots, HR was presented as the 95% confidence interval, the dotted lines indicate the null association, and the Wald’s test was used to determine statistical significance.

Metastatic melanoma includes distant (stage IV) and regional lymph node metastasis (stage III). After validating that the TE and TI signatures were predictors for stage IV melanoma patients as above, we investigated their prognostic values in stage III melanoma patients. We isolated the stage III SKCM samples in TCGA based on the metastatic regions. We found that the distribution of TE-signature and TI-signature scores are highly different. The stage III samples got the highest TE-signature score while the distal metastatic samples got the highest TI-signature score (Figures 4A,B). Then, we calculated the TE-signature and TI-signature scores of samples in two stage III datasets—GSE53118 and GSE54467—and examined their prognostic roles. We found a significant protective association of the TE-signature score with survival (HR = 0.46, *P* = 0.002, Figure 4C) in GSE53118. Adjusting for clinical covariates, including pathological stage at diagnosis, age, and sex, did not substantially change the significant prognostic value of the TE signature we observed (*P* = 0.02, Figure 4D). We were able to repeat this finding in the GSE54467 dataset with the TE signature (HR = 0.38, *P* = 0.003, Figure 4E, *P* = 0.003, Figure 4F). On the contrary, the predictive performance of TI signature was not significant. Therefore, only the TE signature can be used to predict the prognosis of patients with stage III melanoma.

### TE and TI Signatures Provided Additional Prognostic Values Than Clinical Factors

**FIGURE 5:**
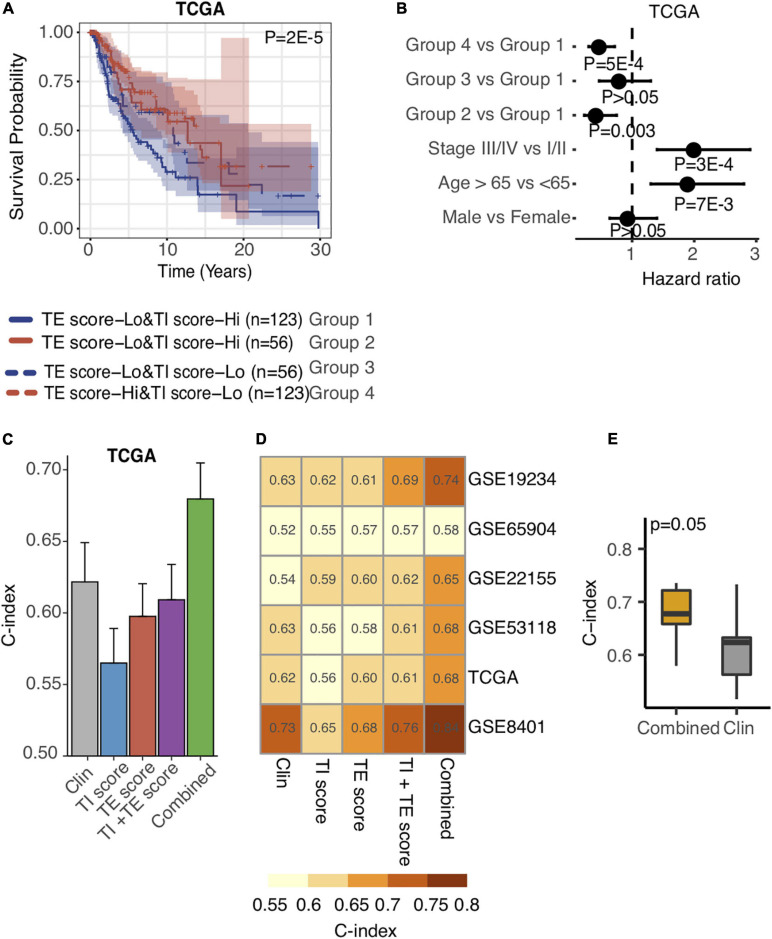
Integration of TE signature and TI signature outperforms prognosis prediction than clinical factors. **(A)** Kaplan–Meier plots depicting the survival distribution for patients in each group. In Kaplan–Meier plots, *p*-values were calculated using the log-rank test, and vertical hash marks indicate censored data. **(B)** Forest plot showing hazard ratios and *p*-values of TE-signature score and several clinical variables estimated by a multivariate Cox regression model. In all forest plots, HR was presented as the 95% confidence interval, the dotted lines indicate the null association, and the Wald’s test was used to determine statistical significance. **(C)** Barplot showing the C-index distribution of using Clinical factors, TI-signature scores, TE-signature scores, combination of TI-signature and TE-signature scores, and combination of all features in predicting prognosis in TCGA data. **(D)** Heat map showing the C-index distribution of features listed in **(C)** across different datasets. **(E)** Boxplot showing the C-index difference between combined prognostic model and clinical factor-derived prognostic model. *P*-value was calculated by the Wilcoxon rank-sum test.

Taking into consideration the distinctive associations of the TE signature and TI signature with patients’ prognosis, we proposed that the integration of TE signature and TI signature could separate patients much better in terms of overall survival. As a result, we examined the predictive performance of TE signature and TI signature and clinical information on the survival outcome of metastatic melanoma patients. First, we separated the samples in the TCGA SKCM datasets into four groups including TE-signature score-Low and TI-signature score-High, TI-signature score-Low and TE-signature score-High, TE-signature score-Low and TI-signature score-Low, and TE-signature score-High and TI-signature score-High. We found that the survival probability of the four groups of samples was significantly different as shown in Figure 5A. As we expected, the group with high TE-signature and low TI-signature scores had the best survival outcome, and the group with low TE-signature and high TI-signature score shaved the worst survival outcome (*P* = 2E-5, Figure 5A). This pattern could still be observed after adjusting for important clinical factors (Figure 5B), highlighting the potential of developing clinical applicable model.

Driven by this, we further conducted a multivariate Cox regression analysis on the TCGA cohort to explore the prediction power differences among TE signature, TI signature, and clinical factors and subsequently developed a prognostic prediction model. Shown in Figure 5C, the model combined all clinical information with TE signature and TI signature achieving the highest prediction performance, measured by C-index. We further quantified the model’s performance on another five independent stage III and stage IV melanoma datasets. The combined model outperformed other models in each independent dataset with the highest C-index = 0.84 being observed in GSE8401 (Figure 5D). As expected, the combined model could significantly improve the prediction of patient’s survival outcome (*P* = 0.05, Figure 5E).

### The TE-Signature Predicted Patients’ Response to Immunotherapy

**FIGURE 6:**
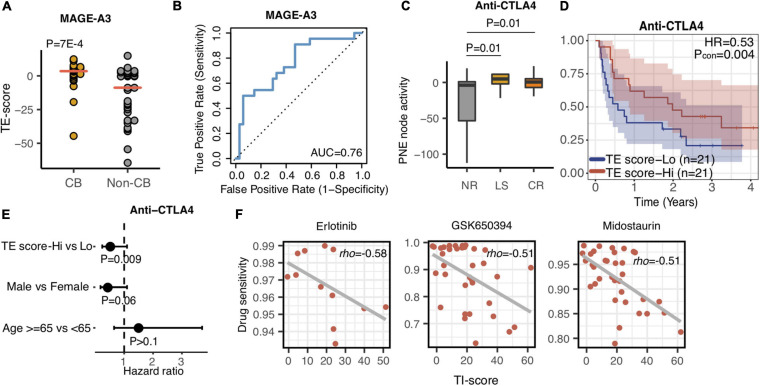
The TE and TI signatures are predictive of drug response. **(A)** Dot plot indicating the TE-signature score difference between responder and non-responder. *P*-value was calculated by Wilcoxon rank-sum test. **(B)** Receiver operating characteristic (ROC) curves for MAGE-A3 therapy response prediction in melanoma patients using the TE-signature score as the predictor. **(C)** Boxplot depicting the TE-signature score difference between different response groups treated with Anti-CTLA4 therapy. *P*-value was calculated by Wilcoxon rank-sum test. **(D)** Kaplan–Meier plots depicting the survival distribution for patients with high (red) and low (blue) TE-signature scores. In Kaplan–Meier plots, *p*-values were calculated using the log-rank test and vertical hash marks indicate censored data. **(E)** Forest plot showing hazard ratios and *p*-values of TE-signature scores and several clinical variables estimated by a multivariate Cox regression model. In all forest plots, HR was presented as the 95% confidence interval, the dotted lines indicate the null association, and the Wald’s test was used to determine statistical significance. **(F)** Scatterplot showing the correlation between TI-signature score and Erlotinib, GSK650394, or Midostaurin drug sensitivity. In all scatterplots, the rho was calculated by spearman correlation.

Various immunotherapy strategies have been developed to save metastatic melanoma patients’ lives, yet many patients do not respond to current immunotherapies. Precisely predicting that the patient cohort may potentially respond to a certain immunotherapy could maximize the benefit of the therapy to the responding patients while minimizing the risks of severe side effects of immunotherapy for the nonresponding patients. MAGE-A3 anti-gen-specific cancer immunotherapy is a tumor vaccine therapy that has been tested in multiple clinical trials (Daud, 2018; Pol et al., 2019). Therefore, we first investigated whether the TE signature can predict the response of patients with metastatic melanoma to this tumor antigen vaccine therapy. We calculated the TE-signature score and compared its difference between the patients who responded or did not respond to the MAGE-A3 immunotherapy. As shown in Figure 6A, there was a significant difference in TE-signature score between two groups of patients (*P* = 7E-4, Figure 6A). Patients who benefited from the MAGE-A3 immunotherapy had significantly higher TE-signature score. An AUC = 0.76 was observed by using the TE-signature score as the predictor (Figure 6B).

In addition to antigen-specific immunotherapy, CBI has achieved great success in treating metastatic melanoma patients (Li et al., 2016; Larkin et al., 2019). We additionally analyzed the association between the TE signature and response to anti-CTLA4 therapy. Using the RECIST criteria, patients were labeled as no response (NR), long survival (LS), and complete response (CR). Shown in Figure 6C, both CR and LS patients had significantly higher TE-signature scores compared to no response patients (*P* = 0.01, CR vs. NR; *P* = 0.01, LS vs. NR). Furthermore, it is not surprising that the TE signature predicted the overall survival in patients treated with anti-CTLA4 therapy and the prediction power remained significant after controlling for clinical factors (*P* = 0.004, HR = 0.53, Figure 6D; *P* = 0.009, Figure 6E).

### The TI Signature Was Associated With Cancer Cell Line Sensitivity to Inhibitors of the MYC Pathway

Given that the TI signature reflected poor clinical outcomes of metastatic melanoma patients (Figures 3, 6), we sought for potential drugs that could inhibit the function of the genes in the TI signature which was annotated as the MYC-related pathway (Figure 2). Using the GDSC database, we examined the association between anticancer drugs and the TI-signature score (Supplementary Table 4). The top three highly correlated anticancer drugs are presented in Figure 6F. Interestingly, all those drugs are reported to be kinase inhibitors and have a certain degree of inhibition on the signaling pathway activated by MYC. Erlotinib and Midostaurin were both FDA-approved tyrosine kinase inhibitors and found to inhibit MYC activity (Suenaga et al., 2013; Basit et al., 2018; Allen-Petersen and Sears, 2019). GSK650394 is a novel serum and glucocorticoid-inducible kinase (SGK) inhibitor and has been reported in treating melanoma cancer in some preclinical studies (Scortegagna et al., 2015).

## Discussion

In this study, we have built a deep-learning-based computational framework to extract tumor-intrinsic features and extrinsic features from the melanoma gene expression data and define a tumor-intrinsic (TI) signature and a tumor-extrinsic (TE) signature. Then, we systematically investigated how TI and TE signatures affect melanoma patients’ prognosis and response to different therapies. To interpret the two signatures, we determined the relative contribution of each gene (bottom node) to them (see Methods). Following that, pathway analyses were performed to identify the underlying pathways. Our results first indicated that the TE signature captured the cytotoxic infiltrating immune cell abundance while the TI signature captured MYC oncogenic pathway activity (Figures 2B–F). Next, we examined the prognostic role of the TE signature and TI signature in metastatic melanoma patients and stage III melanoma patients, respectively (Figures 2–4). Patients with high TE-signature scores would present a better survival outcome in metastatic and stage III melanoma while patients with high TI-signature scores would present a worse survival outcome in metastatic melanoma (Figures 3, 4). Driven by this, we further constructed different prediction models to quantify the prognostic power of the TE signature, TI signature, and clinical factors. As a result, we found the integrative model using the TE signature; the TI signature with a clinical factor achieved a significantly better performance compared with clinical factor-only model (Figure 5). In addition, we showed that the TE signature was predictive of immunotherapy while the TI signature was associated with tyrosine and Ser/Thr kinase inhibitor sensitivity (Figure 6).

While many computational methods have been published to capture the immune cell-associated features in the tumor microenvironment, most of them utilized the linear regression-formulized model to characterize the relationship of immune cell-related genes. Given the complicated gene–gene interactions in the tumors, our method utilized deep learning, integrating both the linear and nonlinear associations between genes, to capture the function of the tumor-extrinsic features (Figures 1, 2). By choosing IHC-measured lymphocyte score positively associated genes, we decomposed the immune microenvironment into 20 nodes which covered different states or types of immune cells. In our analyses, we only chose the most prognostic node, defined as TE signature, to perform the downstream analyses due to its clinical potential (Figure 2). However, the more comprehensive analysis of characterizing other nodes will be interesting in the future.

We performed a similar analysis to capture the tumor-intrinsic feature by using IHC-measured lymphocyte score negatively associated genes. It is interesting to observe that the TI-signature score, which reflects MYC oncogene pathway activity, is strongly associated with prognosis. MYC, known as an important oncogenic regulator, has a high fraction of amplification events in melanoma samples, contributing to the overactivation of the MYC oncogenic pathway (Schaub et al., 2018; Schaafsma et al., 2020). As a result, high MYC activity induces melanoma tumor growth, further leading to metastasis. More importantly, MYC also regulates the immune cell function in the tumor microenvironment. MYC could either directly or cooperate with other oncogenes to regulate the expression of PD-L1 to inhibit the function of immune cells or remodel the tumor microenvironment by recruiting macrophages that promote angiogenesis and reduce T cell infiltration (Casey et al., 2018). It is not surprising that MYC activity is negatively associated with the infiltration level of different immune cells (Figure 2G). Our study highlighted the significance of MYC in melanoma progression from both tumor-intrinsic and -extrinsic perspectives.

The prognostic value of immune cells in metastatic melanoma has been reported many times, and several-immune-cell-based prognostic biomarkers have been proposed. In this work, we selected genes that best reflected the expression of tumor cells and infiltrating immune cells, respectively. These genes were input into autoencoders to extract tumor-intrinsic and -extrinsic features in the form of bottleneck nodes. From them, we selected two representative nodes and defined a TE signature and a TI signature for prognostic prediction. We first validated the prognostic role of TE signature. Surprisingly, our results indicated that the integration of the TE signature and TI signature could further stratify patients into different risk groups. Patients with high TI-signature and low TE-signature scores had the best survival outcome while patients with high TI-signature and low TE-signature scores had the worst survival outcome. The combination prognostic model, which integrates the TE signature, TI signature, and clinical factors, significantly improved the prediction power of clinical factors derived model (Figure 5). These results validated the capability of Autoencoders in denoising and reducing dimensionality for defining prognostic signatures.

Our current model utilized the median score as the cutoff for predicting prognosis because the gene expression profiles from the preclinical cohorts have different scales. To facilitate the clinical application in the future, we could rescale the expression profiles from those preclinical cohorts to build a cohort-independent threshold for clinical practice. One thing to be noted is that the model prediction power was limited by the clinical information that was provided in the public data. In addition to patients’ stage, gender, and Breslow Depth, the surgery information and other treatment information also impact the prognosis in melanoma patients (Bhatia et al., 2015). In the future, with more patient information available, we would like to integrate different clinical information to further improve the prediction accuracy of the combined model.

Targeted immunotherapies have been increasingly used in clinical practice of treating metastatic melanoma patients. MAGE-A3 therapy, a tumor vaccine-based immunotherapy, is still undergoing different clinical trials (Pol et al., 2019). However, several previous clinical trials revealed that MAGE-A3 did not reach the endpoint criteria (Kruit et al., 2005; Dreno et al., 2018). Our results indicated that the TE signature was predictive of MAGE-A3 clinical benefits, which could be further used to guide the design of future clinical trials (Figures 6A,B). In addition to tumor vaccine therapy, immune checkpoint blockade therapy has revolutionarily changed immunotherapy and significantly improved overall survival (Larkin et al., 2019). In our results, TE signature could predict anti-CTLA4 response (Figure 6C). Patients with high TE-signature scores were more likely to be responders and had a better survival outcome (Figure 6D). This result raised the potential of using the TE-signature score as a biomarker for anti-CTLA4 response prediction. In our current analysis, only regular clinical information, including patients’ age, gender, and stage, was provided. The efficacy of immunotherapy was also affected by other treatment strategies. For example, chemotherapy administered after immunotherapy might improve the immunotherapy response (Fridlender et al., 2010; Peng et al., 2015). In the future, with such treatment information being released, the prediction accuracy of using the TE signature could be further enhanced.

In the previous section, we mentioned the importance of MYC from both tumor-extrinsic and -intrinsic sides. Inhibiting MYC in melanoma will bring a reduction in tumor proliferation and potentially remodel the tumor microenvironment into immune hot, leading to the increased sensitivity of immunotherapy. Using the GDSC database, we identified that Erlotinib and Midostaurin have inhibitory roles for MYC pathway activity (Figure 6F). Erlotinib and Midostaurin were both FDA-approved tyrosine kinase inhibitors and found to repress MYC activity (Suenaga et al., 2013; Basit et al., 2018; Allen-Petersen and Sears, 2019). Interestingly, several clinical trials are ongoing for testing the efficacy of Erlotinib combined with immune-checkpoint blockade therapy (Liang et al., 2018). Our analysis highlighted the potential clinical usage of MYC inhibitors in treating metastatic melanoma patients (Singleton et al., 2017).

In summary, we developed a computational framework to capture the tumor-extrinsic and -intrinsic features in melanoma patients. The two TE- and TI-signature scores we calculated as the representatives of tumor cell feature and immune cell feature are powerful in predicting patient prognosis and response to different treatments. The computational framework could be readily extended to other cancer types.

## Materials and Methods

### Dataset Collection

The TCGA melanoma RNA-seq data were downloaded from Firehose^1^ (Supplementary Table 1), containing gene expression profiles of 358 metastatic patients. Gene expression values were calculated and normalized by using the RNA-Seq by Expectation-Maximization (RSEM) Algorithm (Li and Dewey, 2011). The clinical information of TCGA melanoma samples was also retrieved from Firehose (see text footnote 1). The information included the patients’ age, gender, pathological stage at diagnosis, location of the metastatic tumor, Breslow thickness, lymph node stage, and metastatic stage.

Six additional microarray data sets were used for metastatic melanoma and stage III melanoma prognosis analysis. These data were downloaded from the Gene Expression Omnibus (GEO) database with accession numbers GSE65904 (*n* = 214), GSE54467 (*n* = 79), GSE53118 (*n* = 79), GSE22155 (*n* = 54), GSE8401 (*n* = 47), and GSE19234 (*n* = 44) (Xu et al., 2008; Bogunovic et al., 2009; Jönsson et al., 2010; Mann et al., 2013; Cirenajwis et al., 2015; Jayawardana et al., 2015). GSE65904 and GSE19234 contained disease-specific survival time (DSS) and survival time information after recurrence, respectively, while TCGA-SKCM, GSE54467, GSE53118, GSE22155, and GSE8401 data sets contained overall survival time (OS) information. GSE53118 and GSE54467 provided the survival information for patients with stage III melanoma.

Two datasets were used for immunotherapy response analysis. The treatment information of MAGE-A3 immunotherapy is included in the GSE35640 dataset. It provided the gene expression profiles of a total of 56 patients, among which 34 had no responses and 22 had clinical benefits (Ulloa-Montoya et al., 2013). The anti-CTLA4 immune checkpoint blockade therapy dataset was downloaded from the Database of Genotypes and Phenotypes (dbGaP) under accession number phs000452 (Van Allen et al., 2015). Raw read files were aligned to the GRCh37 human genome assembly using the TopHat v2.1.0 (Kim et al., 2013), and the gene expression was calculated using the Cufflinks v2.2.1 (Trapnell et al., 2012). In total, 42 treatment-naive tumor sample patients were sequenced.

The Genomics of Drug Sensitivity in Cancer (GDSC) dataset was downloaded from the GDSC database^2^ for anticancer drug sensitivity testing (W. Yang et al., 2013). It provided a baseline gene expression for a total of 987 cell lines, including with 38 melanoma cell lines, with the corresponding sensitivity to 251 drugs. Drug sensitivity was represented as Area Under the Curve for the fitted model (AUC), with lower values indicating higher sensitivity to a drug (i.e., lower IC50 values).

The genomic characteristics of TCGA melanoma samples were calculated based on the MAF file and DNA sequencing map downloaded from Firehose (see text footnote 1). Specifically, tumor mutation burden (TMB) was represented as the total number of non-silent somatic mutations in a given TCGA melanoma sample. The copy number variation burden (CNV burden) was calculated using the following equation:

(1) $$C\,N\,V-b\,u\,r\,d\,e\,n=\frac{\sum_{j=1}^{m}\left|l\,o\,g_{2}\,\left(\frac{c_{j}}{2}\right)*f_{j}\right|}{N}$$

where *C*_*j*_ and *f*_*j*_ represent the copy number and the size of the DNA fragment j in the sample; m is the total number of abnormal fragments in the genome, and N is the size of the human genome. For a normal diploid genome, the CNV burden is zero. A higher CNV burden indicates a higher level of copy number variation of the genome.

### Gene Expression Decomposition Based on Autoencoder

We applied an autoencoder model to decompose gene expression data for metastatic melanoma samples using the RNA-seq from TCGA. An autoencoder is a type of artificial neural network consisting of two components: an encoder that gradually reduces the input gene expression data into a small number of representative nodes and a decoder that reconstructs the original input (Chen et al., 2018; Way and Greene, 2018; Supplementary Figure 1). The configuration of the Autoencoder is shown in Supplementary Figure 1; we used two layers for Encoder and Decoder with each layer containing 400 and 100 nodes, respectively. By minimizing the deviation between the reconstructured and the input data, Autoencoder achieves dimensionality reduction using the 20 representative nodes while filtering out noises (Supplementary Figure 1). As shown in Figure 1, the main steps are elaborated below.

First, TCGA metastatic melanoma RNA-seq data were log transformed and converted into z-scores by subtracting the mean and then dividing the standard deviations of genes across all samples. In order to capture both tumor cell-intrinsic and -extrinsic signals, we selected the top 1000 genes that had the highest positive correlations with lymphocyte infiltration scores ($G_{H}^{{}^{\prime }})$ and the top 1000 genes that had the highest negative correlations $(G_{L}^{{}^{\prime }}$). Lymphocyte infiltration scores were calculated based on IHC staining results from TCGA (Cancer Genome Atlas Network, 2015).

Second, for both of the two gene expression sub-matrices ($G_{H}^{{}^{\prime }}$ and $G_{L}^{{}^{\prime }}$), an Autoencoder model was used to identify 20 informative “hidden” nodes that best capture the whole expression sub-matrices. Autoencoder could integrate both linear and nonlinear structures in the gene expression data and therefore more correctly capture complex gene–gene interactions. Specifically, the configuration of the AutoEncoder model is shown in Supplementary Figure 1. There were 1000 nodes of the input layer, corresponding to the gene expression after screening, and then compressed to 400, 100, and 20 nodes in the following layers, and then gradually reconstructed. Each layer of the model is fully connected, and each hidden layer is followed by a rectified linear unit (ReLU) activation function, which is defined as follows.

(2) $$R\,e\,L\,U\,\left(x\right)=\left\{ \begin{array}{ccc} x\; & i\,f & x\geq 0\\ 0\; & i\,f & x<0 \end{array}\right.$$

In order to train the model, we chose the regularized square loss as the objective function, as shown in equation 5.

(3) $$L=\sum_{i=1}^{n}\varepsilon \,\left(i\right)+{||w||}^{2}=\frac{1}{2}\,\sum_{i=1}^{n}{||x-D_{\theta }\,\left(E_{\theta }\,\left(x\right)\right)||}^{2}+\lambda \,{||w||}^{2},$$

where n denotes the number of samples and *E*_θ_ and *D*_θ_ represent the encode and decode functions, respectively. *w* represents the learnable weight of the AutoEncoder model. λ is the hyperparameter controlling the proportion of the regularization term. We chose a stochastic gradient descent (SGD) optimization method to train the model and to obtain the optimal weight ***w***. The compressed features *F*_*H*_ and *F*_*L*_ corresponding to $G_{H}^{\prime }$ and $G_{L}^{\prime }$ can be obtained by the two well-trained AutoEncoder models, as shown in equations 6 and 7.

(4) $$F_{H}=E_{\phi_{1}}\,\left(G_{H}^{{}^{\prime }}\right)$$

(5) $$F_{L}=E_{\phi_{2}}\,\left(G_{L}^{{}^{\prime }}\right)$$

where *F*_*H*_ and *F*_*L*_ are two matrices with 20 columns; each row represents a sample, and each column represents a feature compressed by the AutoEncoder model. The performance of the autoencoder model was measured by the R square between the fitted gene expression and the real gene expression. We also tried different numbers of nodes in the bottleneck layer and found the comparable performance.

Finally, from the compressed features *F*_*H*_ and *F*_*L*_, we selected a feature that best correlated with patient prognosis in TCGA metastatic melanoma samples. Since the two selected features, respectively, capture tumor cell-intrinsic and -extrinsic features, we denoted them as tumor-intrinsic (TI) and tumor-extrinsic (TE) signatures.

### Calculation of TE- and TI-Signature Scores in Tumor Samples

For a given melanoma gene expression dataset, we first utilized a Z-score transformation to convert the expression profile to a relative expression profile. We then separated the relative expression profile into two profiles, containing $G_{H}^{{}^{\prime }}$ and $G_{L}^{{}^{\prime }}$ genes, respectively. For each patient in the relative expression profile, we applied the Autoencoder models trained in the TCGA-SKCM metastatic dataset and acquired the corresponding TE- and TI-signature scores according to equations 4 and 5.

### Survival Analysis

Cox proportional hazard models were used to investigate the association between signature scores (calculated based on the TE signature or TI) and patient prognosis. Patient samples were dichotomized into two groups by using the median score as the cutoff value. Univariate Cox regression models were used to determine the association between the dichotomized scores and patient survival. To compare survival between the two groups, Kaplan–Meier plots were used for visualization. The difference between the survival times of different groups was compared by a log-rank test. The multivariate Cox regression model was used to estimate the association between signature scores and patient survival while considering important clinical variables such as age, sex, Breslow score, and tumor stages.

The Kaplan–Meier estimator was implemented in the survival R package. Specifically, the “coxph” function was used to construct Cox proportional hazard models. The “survfit” function was used to generate Kaplan–Meier survival curves. The “survdiff” function was used to statistically compare the difference between survival curves.

### Gene Weight Calculation

After model training, we obtained the weights of each layer in TE and TI signature-associated Autoencoder models. The genes with more contributions to the signature tend to have higher weights. The weighted sum of all the possible combinations between each gene and the corresponding signature node (the TE signature-17th node in the *F*_*H*_ and the TI signature-7th node in *F*_*L*_) can be viewed as the contribution score. The score is defined as follows.

(6) $$\text{GWH}\,\left(\text{i}\right)=\sum_{\begin{array}{cc} j=1:400\; & \\ k=1:100\; & \end{array}}w_{i,j}^{\left(1\right)}\cdot w_{j\,k}^{\left(2\right)}\cdot w_{k,17}^{\left(3\right)}$$

(7) $$\text{GWL}\,\left(\text{i}\right)=\sum_{\begin{array}{cc} j=1:400\; & \\ k=1:100\; & \end{array}}w_{i,j}^{\left(1\right)}\cdot w_{j\,k}^{\left(2\right)}\cdot w_{k,7}^{\left(3\right)}$$

where $w_{a,b}^{\left(c\right)}$ represents the weight between the *b*th node of the *c*th hidden layer and the *a*th node of the prior layer. So GWH (i) and GWL (i) represent the importance score of the *i*th gene in the TE and TI signature, respectively.

### Pathway Analysis

Based on the weight profile that each gene contributes to the node, we performed pre-rank Gene Set Enrichment Analysis using the fgsea R package (Korotkevich et al., 2019). For calculating the specific pathway activity in melanoma patients, Gene Set Variation Analysis was used for integrating the expression profile with the MsigDB C2 pathway database (Subramanian et al., 2005) through GSVA R package (Hänzelmann et al., 2013).

## Data Availability Statement

The original contributions presented in the study are included in the article/Supplementary Material, further inquiries can be directed to the corresponding author/s.

## Author Contributions

CC contributed to conception and design. CC, YS, YD, and YZ contributed to the development of methodology and analysis and interpretation of the data. CC, YD, and YZ contributed to the writing–review, and/or revision of the manuscript. All authors contributed to manuscript revision, read, and approved the final manuscript.

## Conflict of Interest

The authors declare that the research was conducted in the absence of any commercial or financial relationships that could be construed as a potential conflict of interest.

## Footnotes

1 http://gdac.broadinstitute.org/

2 https://www.cancerrxgene.org
