# Supplementary figures and images for: AutoEncoder-Based Computational Framework for Tumor Microenvironment Decomposition and Biomarker Identification in Metastatic Melanoma
Source: Front Genet. 2021 May 27;12:665065. doi: 10.3389/fgene.2021.665065 (PMC8191580; doi:10.3389/fgene.2021.665065)

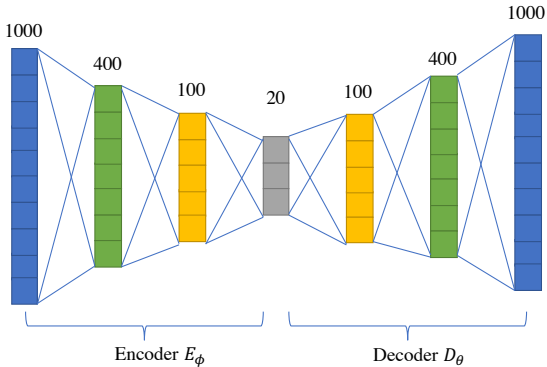

Supplement: Supplementary file 1 [file Data_Sheet_1.PDF]
